# Valence Bond Insights into the H-Abstraction Barrier in Cytochrome P450

**DOI:** 10.3390/molecules30102242

**Published:** 2025-05-21

**Authors:** Enhua Zhang, Hajime Hirao

**Affiliations:** Warshel Institute for Computational Biology, School of Medicine, The Chinese University of Hong Kong, Shenzhen 518172, China; 223055007@link.cuhk.edu.cn

**Keywords:** cytochrome P450, iron(IV)–oxo, valence bond theory, hydrogen abstraction, correlation diagram

## Abstract

The valence bond (VB) framework is widely recognized as a powerful tool for elucidating the electronic origins of activation energy barriers in chemical reactions. We employed ab initio VB calculations to investigate the hydrogen abstraction (H-abstraction) barrier in cytochrome P450 enzymes (P450s), using a simplified model in which an oriented external electric field (OEEF) was applied to efficiently capture the electronic effects of the equatorial porphyrin and proximal thiolate ligands on the iron(IV)–oxo unit in compound I (Cpd I). Methane (CH_4_) was used as the model substrate. The VB-calculated barrier height, evaluated with this simplified model, qualitatively reproduced the barrier predicted by density functional theory (DFT) calculations using a more complete active-site model. Additionally, by examining the weights and diagonal elements of the Hamiltonian matrix for different VB structures along the reaction coordinate, we identified key VB structures—including covalent and ionic configurations representing the C–H and O–H bonds—that contribute significantly to the electronic origin of the barrier height. The mixing of these distinct VB structures leads to resonance stabilization, which is maximized at the transition state.

## 1. Introduction

**Scheme 1:**
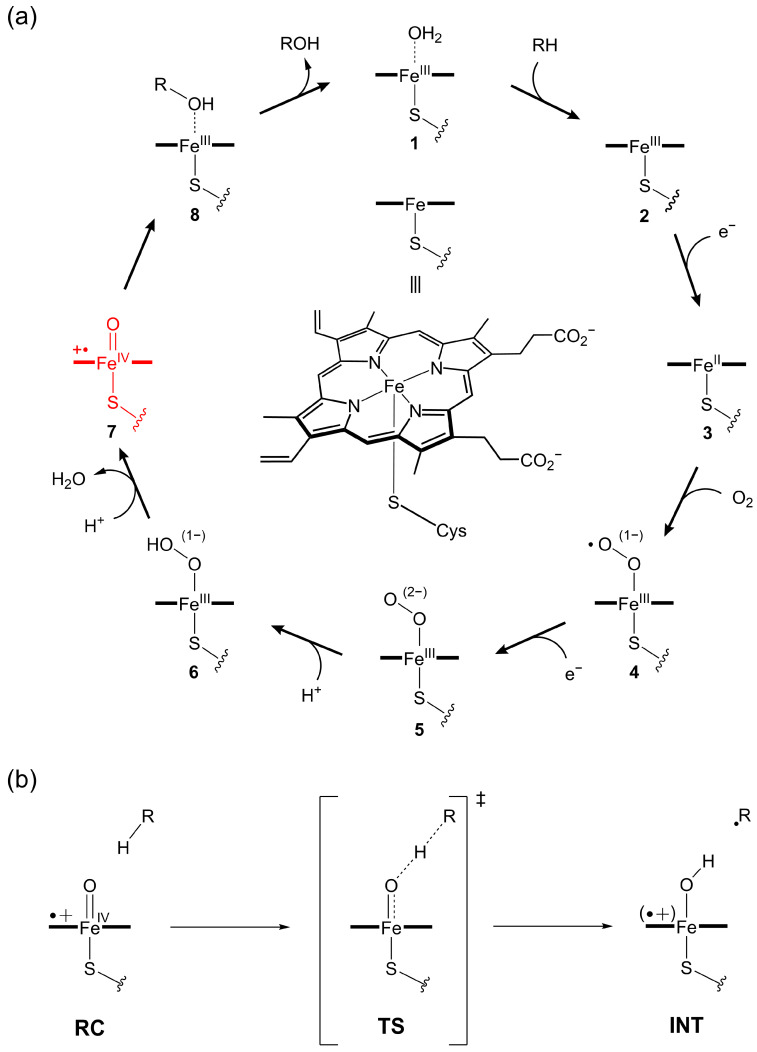
(**a**) Catalytic cycle of P450s with Cpd I highlighted in red. (**b**) Schematic illustration of the H-abstraction from an alkane (R–H) by Cpd I. During this process, an electron from the substrate migrates either to the iron center or to the porphyrin ligand, depending on the reaction pathway.

Hydrogen abstraction (H-abstraction) from alkanes is a key step in substrate activation catalyzed by cytochrome P450 enzymes (P450s) [1]. This process occurs in diverse organisms, from bacteria (e.g., camphor hydroxylation by P450cam [2]) to humans (e.g., paclitaxel hydroxylation by CYP2C8 [3]), and contributes to important physiological and pharmacological outcomes. P450-catalyzed alkane hydroxylation typically proceeds via a two-step mechanism in which H-abstraction is followed by an oxygen rebound step. The H-abstraction step is generally considered the rate-limiting step [4,5,6] and is mediated by a high-valent iron(IV)–oxo π-cation radical species, compound I (Cpd I), which forms in the catalytic cycle and serves as the primary oxidant (Scheme 1a) [7,8,9]. Similar H-abstraction processes also occur in both enzymatic and synthetic systems [10,11,12,13], underscoring the broader relevance of this fundamental transformation in oxidation chemistry.

Over the past two decades and beyond, the mechanistic aspects of H-abstraction by iron(IV)–oxo species have been extensively investigated. In this effort, density functional theory (DFT) [14,15,16,17,18,19,20] and DFT-based hybrid quantum mechanics/molecular mechanics (QM/MM) methods [2,3,21,22,23,24,25,26,27], which provide a favorable balance between accuracy and computational efficiency, have served as the primary tools for computational investigation. In addition, ab initio quantum chemical calculations have also been employed to study H-abstraction processes, providing higher accuracy in select cases at the cost of increased computational demand [28,29,30]. These molecular orbital (MO)-based computational approaches have yielded valuable insights into the geometries, potential energy surfaces, and electronic structures associated with H-abstraction, substantially advancing our understanding of the reactivity and selectivity of high-valent iron–oxo species in both enzymatic and synthetic systems.

Despite this progress, a key question remains regarding the fundamental origin of H-abstraction reactivity. Although MO-based approaches are widely used and computationally efficient, they do not always clearly reveal the electronic factors responsible for activation barriers. This limitation arises from the inherently delocalized nature of MOs, which can obscure localized electronic descriptions of chemical bonding. In contrast, valence bond (VB) theory provides a more localized and chemically intuitive framework for analyzing bonding interactions. Furthermore, conceptual tools such as state correlation diagrams and configuration mixing diagrams enable visualization of how different electronic structures contribute to the rise and fall of the potential energy along the reaction coordinate, particularly during bond-breaking and bond-forming events [31,32,33,34,35]. These tools help identify key electronic configurations that dominate in the reactant, transition state, and product regions, thereby offering deeper insight into the origins of activation energy barriers. VB-based correlation diagram approaches have been successfully applied to rationalize the reactivity of a wide range of organic transformations and, more recently, have been used to qualitatively—but effectively—interpret reactivity trends in P450s [34,35].

However, to the best of our knowledge, such diagrams have not yet been generated for P450 systems using ab initio VB calculations. This is largely due to the significantly higher computational cost of VB methods relative to DFT and other MO-based approaches. Several VB studies relevant to P450 chemistry have recently been conducted by our group. For example, our VB study on the ferrous–CO complex demonstrated that π backdonation plays a more dominant role in bonding than σ donation [36]. Furthermore, we found that VB structures representing different bonding patterns act synergistically to strengthen bonding through resonance. In a more recent study on iron(IV)–oxo bonding in Cpd I, we identified key VB structures contributing to the bonding interaction and showed that its bonding character differs substantially from that of a conventional double bond, despite its common depiction as Fe(IV)=O [37]. We also explored the quantum mechanical origins of local interactions occurring at the heme in P450–inhibitor complexes using various theoretical tools [38,39,40]. Together, these studies have advanced our understanding of local chemical bonding in P450 systems. However, the application of ab initio VB theory to chemical reactions in P450s, such as substrate oxidation, has not yet been explored.

In this study, we take an initial step toward addressing this gap by applying ab initio VB calculations to a simplified P450–substrate model system that incorporates an oriented external electric field (OEEF) to mimic the electronic environment surrounding the iron(IV)–oxo unit. The use of an OEEF is motivated by our recent findings, which demonstrated that a properly oriented and tuned field can effectively reproduce the electronic effects of the equatorial porphyrin and proximal thiolate ligands [41]. Electric fields around iron–oxo species have been studied both for their influence on reactivity trends and for understanding how protein environments contribute to such fields [42,43,44,45,46,47,48]. In this study, by replacing explicit ligands with a strategically oriented electric field, we aim to reduce the size of the model system while preserving the essential electronic features of the enzymatic environment. Our investigation focuses on the H-abstraction step in P450-catalyzed alkane hydroxylation (Scheme 1b), which involves three key stationary points along the reaction coordinate: the reactant complex (RC), the transition state (TS), and the intermediate (INT). This reaction step, generally regarded as rate-limiting in alkane hydroxylation, offers a meaningful and tractable target for VB analysis.

## 2. Results and Discussion

### 2.1. DFT Calculations

We began by performing DFT calculations on the H-abstraction step shown in Scheme 1b, using a simplified model system without OEEFs. In this model, a porphine ring and a hydrosulfide anion (HS^−^) were used to represent the equatorial and proximal ligands, respectively. Methane (CH_4_) was chosen as the model alkane substrate due to its structural simplicity. Methane has also been employed in several previous computational studies on P450 Cpd I reactivity [49,50,51]. The geometries of the stationary points along the reaction pathway (RC, TS, and INT) were optimized using the above-described model and are denoted as RC_0_, TS_0A_/TS_0B_, and INT_0A_/INT_0B_. The subscripts A and B indicate two distinct electronic pathways, referred to as Paths A and B, which are described in detail below.

**Figure 1:**
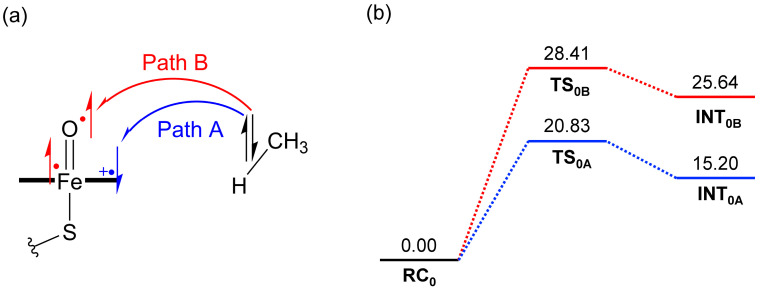
(**a**) Schematic illustration of two possible electronic pathways (A and B) of H-abstraction by P450 Cpd I. (**b**) DFT-calculated energy profiles (in kcal/mol) for Path A (blue) and Path B (red).

Previous DFT studies have identified two distinct electronic pathways (Paths A and B) for H-abstraction by P450 Cpd I in the doublet spin state (Figure 1a) [15]. In Path A, one electron from the substrate shifts to the a_2u_-type porphyrin orbital, whereas in Path B, the electron shift occurs to one of the π* orbitals of the iron(IV)–oxo moiety. Path A is more commonly emphasized in DFT and QM/MM studies due to its generally lower activation barrier and, consequently, greater kinetic favorability. Consistent with this trend, the DFT-calculated barrier for Path A is 20.83 kcal/mol (Figure 1b), which is lower than that for Path B (28.41 kcal/mol). Nonetheless, Path B has also been shown to exhibit comparable stability to Path A in several P450 systems and is frequently considered in mechanistic investigations [22]. The calculated barriers are somewhat higher than those obtained previously for the reaction between Cpd I and camphor using a dispersion-corrected B3LYP method [20], which can be attributed to the more pronounced dispersion stabilization at the TS in the case of camphor, as well as the inherently lower reactivity of CH_4_. Importantly, because Path A involves an electron shift to a porphyrin orbital—a feature not adequately captured in the current VB model lacking ligands—we focus exclusively on Path B in this study. To establish a reference point for our subsequent VB analysis, we first investigated Path B using DFT. The energy of the resulting intermediate (INT_0B_) was calculated to be 25.64 kcal/mol above the RC, indicating that the H-abstraction step is highly endothermic.

### 2.2. VB Calculations

**Scheme 2:**
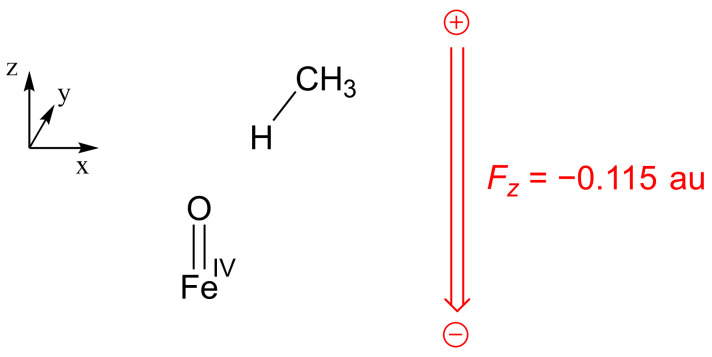
Simplified model system and the applied electric field used in the VB calculations. The Fe–O bond is aligned along the *z*-axis.

For the VB calculations, we employed a minimal model consisting of [Fe(IV)O]^2+^ and CH_4_. To emulate the electronic influence of the equatorial porphyrin and proximal thiolate ligands, an OEEF (*F_z_*) with a magnitude of −0.115 au was applied along the Fe–O bond axis in the downward direction (Scheme 2). This specific field strength and orientation were based on our recent VB study, which demonstrated that such an OEEF can effectively reproduce ligand-induced electronic effects in a larger Cpd I model [41]. More specifically, the weights of different VB structures in a Cpd I model containing four equatorial NH_3_ and one proximal HS^−^ ligand (“Model III”) were well reproduced using this minimal ligand-free model with this particular OEEF. VB models corresponding to the RC, TS, and INT in Path B, denoted as RC_1_, TS_1B_, and INT_1B_, respectively, were constructed using the DFT-optimized geometries of RC_0_, TS_0B_, and INT_0B_ as references. Additional details regarding model construction are provided in the Section 3.

**Scheme 3:**
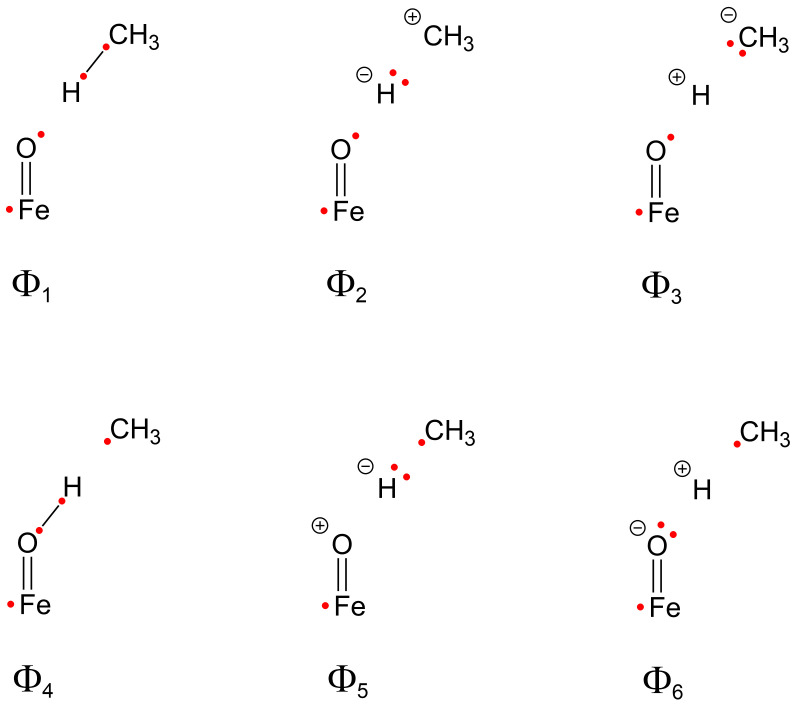
Six VB structures selected for VB analysis. Red dots represent active electrons. The iron–oxo unit was not subjected to explicit VB treatment and is represented as a double bond. For more detailed discussions of Fe–O bonding, see Refs. [37,41].

Although accurately describing the wave function for even this simple model requires a large number of VB structures, the primary objective of this study is to obtain a qualitative, first-step understanding of electronic factors governing the H-abstraction process while minimizing computational cost. In line with this goal, we selected a representative subset of VB structures, denoted as Φ_1_–Φ_6_, for inclusion in our VB calculations (Scheme 3). These structures were chosen to capture the essential bonding patterns and electron reorganization associated with the H-abstraction step. Additional details regarding the selection of VB structures are provided in the Section 3.

**Figure 2:**
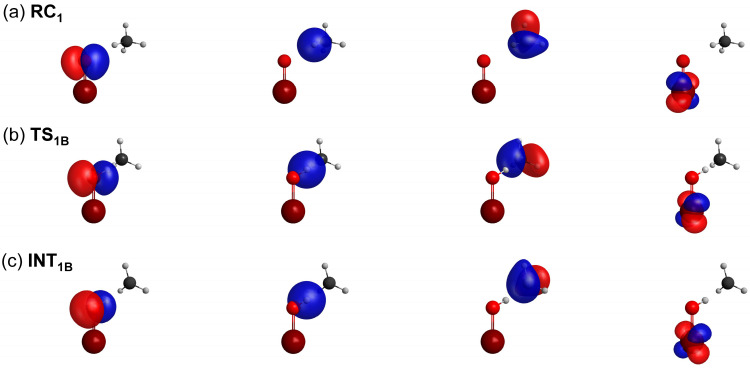
The four active orbitals for (**a**) RC_1_, (**b**) TS_1B_, and (**c**) INT_1B_, as obtained from VBSCF calculations.

Figure 2 presents the four active orbitals obtained from valence bond self-consistent field (VBSCF) calculations for the RC_1_, TS_1B_, and INT_1B_ models. Overall, all orbitals exhibit strong localization on individual atomic centers, in clear contrast to delocalized MOs. However, notable changes in orbital orientation are observed along the reaction coordinate. In RC_1_, where CH_4_ has not yet begun to engage in significant bond formation or cleavage, the orbitals largely retain the character of isolated atomic orbitals, with minimal hybridization. In contrast, as the hydrogen atom approaches the oxo ligand in the transition state (TS_1B_) and intermediate (INT_1B_), the orbitals reorient, with their lobes aligning more closely along the O–H–C axis. This reorientation enhances orbital overlap and facilitates the electronic reorganization required for H-abstraction.

### 2.3. Comparison Between DFT and VB Energies

We first compared the relative energies of RC, TS, and INT along Path B, as obtained from both DFT and VB calculations (Table 1). The DFT-calculated barrier height was 28.41 kcal/mol, whereas the corresponding barrier heights from VBSCF and breathing orbital valence bond (BOVB) calculations were somewhat higher, at 43.46 and 39.69 kcal/mol, respectively. In contrast, the energy of INT was lower in the VB calculations, 11.52 kcal/mol with VBSCF and 10.19 kcal/mol with BOVB, compared to 25.64 kcal/mol in the DFT result. These findings indicate that the VB methods overestimate the activation barrier while underestimating the reaction energy for H-abstraction. Despite these quantitative discrepancies, the VB calculations correctly capture the key energetic feature of the H-abstraction step, namely its endothermic character. This qualitative agreement suggests that, despite the simplicity of the VB model, it retains the essential electronic features governing the reaction energetics.

### 2.4. Weights of VB Structures

To track how the relative contributions of individual VB structures evolve throughout the H-abstraction process, we monitored the VB structure weights *W_K_* (*K* = 1–6) obtained from BOVB calculations (Table 2). Specifically, structures Φ_1_ through Φ_3_ provide a complete VB description of the C–H bond, comprising one covalent structure (Φ_1_) and two ionic structures (Φ_2_ and Φ_3_). The sum of their weights is denoted as *W*_CH_. Likewise, *W*_OH_ denotes the combined weights of Φ_4_ through Φ_6_, which together describe the O–H bond (Scheme 3). In the wave function of RC_1_, Φ_1_ is the dominant contributor, with a weight of 68.30%, representing a Heitler–London-type covalent C–H bond in methane. The ionic structure Φ_3_, which features an anionic CH_3_ fragment, also contributes significantly (27.43%). In contrast, Φ_2_, which represents a cationic CH_3_ fragment, has only a minor contribution (4.27%). Collectively, these three structures (Φ_1_–Φ_3_) account for 100.00% of the total wave function at this stage, while the remaining structures (Φ_4_–Φ_6_) contribute 0.00%, consistent with the absence of O–H bond formation in the reactant state.

At TS_1B_, Φ_4_ exhibits the highest weight (35.98%), followed closely by Φ_6_ (35.06%). Interestingly, although the total weight of the O–H bond-related structures (*W*_OH_ = 74.70%) dominates, the contribution from the C–H bond-related structures (*W*_CH_ = 25.30%) remains appreciable. This yields a *W*_OH_ to *W*_CH_ ratio of approximately 3:1. The greater contribution from O–H bond-related structures indicates that the hydrogen atom is closer to the oxo ligand than to the carbon, consistent with a late transition state. It is also notable that the ionic structures Φ_2_ and Φ_5_, both involving an anionic hydrogen, contribute only marginally at this point along the reaction coordinate.

At INT_1B_, the calculated weights further confirm the progression of H-abstraction; *W*_CH_ drops to 1.30%, while *W*_OH_ increases sharply to 98.70%. This shift indicates that the hydrogen atom has been almost completely transferred from CH_4_ to the iron(IV)–oxo species and that the O–H bond is essentially fully formed. Among the O–H bond-related structures, Φ_4_, which represents a covalent O–H bond, is the most dominant, contributing 57.34%. Its ionic counterpart involving an anionic hydrogen fragment, Φ_5_, remains a minor contributor (3.48%). In contrast, the other ionic structure, Φ_6_, which features a cationic hydrogen fragment, exhibits a substantial weight of 37.89%. The relatively large contribution from Φ_6_ at INT_1B_ suggests that the O–H bond possesses considerable ionic character—greater than that of the C–H bond in RC_1_.

**Figure 3:**
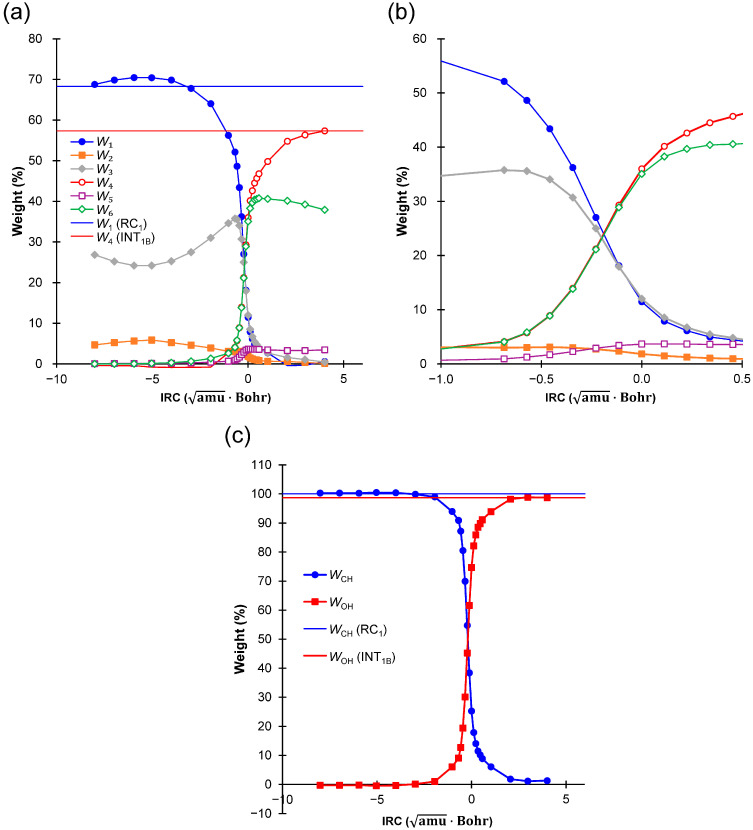
(**a**) Weights (in %) of Φ_1_–Φ_6_, plotted against the IRC. The blue and red horizontal lines represent the weight of Φ_1_ for RC_1_ and that of Φ_4_ for INT_1B_, respectively. (**b**) Enlarged view of the graph near the TS region. (**c**) Evolution of *W*_CH_ and *W*_OH_ along the IRC. The blue and red horizontal lines represent *W*_CH_ at RC_1_ and *W*_OH_ at INT_1B_, respectively.

Building upon these analyses, we further calculated the weights of different VB structures along the intrinsic reaction coordinate (IRC) [52]. The IRC was computed using the DFT model, and smaller VB models were subsequently constructed based on the corresponding DFT geometries. Thus, the IRC values correspond specifically to the DFT model and not the VB model itself. Figure 3a shows the weights of individual VB structures at various points along the IRC. On the reactant side, Φ_1_ exhibits the highest weight, consistent with the RC_1_ result shown in Table 2. Although the weight of Φ_1_ initially shows a slight increase, substantial electronic reorganization occurs in the vicinity of the TS, where its weight sharply declines as the system progresses toward the intermediate region. At the same time, the weight of Φ_4_ begins to increase, eventually reaching 57.34% at INT_1B_. These observations indicate that the covalent VB structures Φ_1_ and Φ_4_ are the primary contributors to the C–H bond-breaking and O–H bond-forming during H-abstraction. In addition, the ionic structures Φ_3_ and Φ_6_ also exhibit notable contributions along the reaction coordinate. As previously noted, *W*_3_ has a relatively large value of 27.43% at RC_1_. Interestingly, this weight does not decrease monotonically; instead, it increases further near the TS before dropping sharply to nearly zero as the system approaches the intermediate. A similar trend is observed for *W*_6_, which rises steeply around the TS and then gradually declines as the system reaches INT_1B_. In contrast, the remaining VB structures, Φ_2_ and Φ_5_, maintain negligible contributions throughout the entire reaction path.

As shown in the plots of VB weights (Figure 3a), significant electronic reorganization occurs within a very narrow range around the TS. To better visualize this reorganization, we expanded the graph to focus on the region between −1.0 and 0.5 amu^1/2^•Bohr (Figure 3b). The crossing of weight curves occurs at approximately −0.2 amu^1/2^•Bohr, slightly preceding TS_0B_ (0.0 amu^1/2^•Bohr) in the DFT model. As we will discuss later, the peak of the total energy calculated using VB also appears near −0.2 amu^1/2^•Bohr, indicating a slight shift in the reaction coordinate between the DFT and VB models. Around this point, the relative dominance of *W*_CH_ and *W*_OH_ reverses (Figure 3c), reflecting the cleavage of the C–H bond and the formation of the O–H bond. The data in Figure 3 also show that the contribution to *W*_CH_ arises primarily from *W*_1_ and *W*_3_, while *W*_OH_ is dominated by *W*_4_ and *W*_6_.

**Figure 4:**
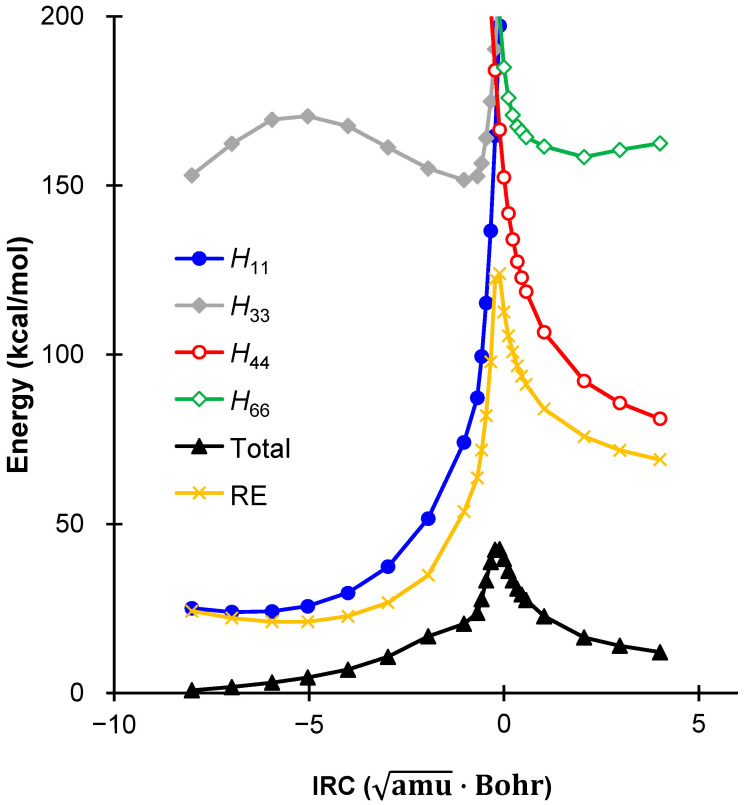
Total energy, diagonal Hamiltonian matrix elements (including nuclear repulsion), and resonance energy (in kcal/mol) plotted along the IRC and reported relative to the total energy of RC_1_.

In addition to weight analysis, we also collected and plotted the diagonal Hamiltonian matrix elements for the VB structures (*H_KK_*) from BOVB calculations along the IRC, as well as the corresponding total energies (Figure 4). The *H_KK_* values for Φ_2_ and Φ_5_ were significantly higher than the range displayed in this figure and were therefore omitted from the plot. Notably, the total energy exhibits a peak around −0.2 amu^1/2^•Bohr, indicating that the TS in the VB calculations occurs slightly earlier than the TS obtained from the DFT calculations. Despite this minor deviation, the VB model reasonably reproduces the shape of the potential energy profile. To further investigate the interactions between VB structures, we also calculated the resonance energy (RE) at each point along the IRC. Here, RE is defined as the difference between the lowest *H_KK_* value and the total energy. Consistent with the weight analysis, Φ_1_ and Φ_4_ exhibit low energies and are the dominant contributors on the RC and INT sides, respectively, with the most significant variations occurring in a narrow region around the TS. A reversal in the relative importance of Φ_1_ and Φ_4_ occurs just before the TS, around −0.2 amu^1/2^•Bohr along the IRC. The RE value was smaller at RC than at INT, which correlates with the greater dominance of Φ_1_ at RC (68.30%, Table 2) than Φ_4_ at INT (57.34%, Table 2). The lower weight of the dominant VB structure at INT suggests a relatively greater contribution from other VB structures, implying enhanced configuration mixing. Interestingly, near the TS, where Φ_1_, Φ_3_, Φ_4_, and Φ_6_ exhibit comparable stabilities and weights, their substantial mixing leads to the highest RE value, exceeding 120 kcal/mol. Thus, resonance stabilization reaches a maximum at the TS along the reaction coordinate, effectively mitigating the steep rise in total energy in this region.

### 2.5. Spin Density

**Figure 5:**
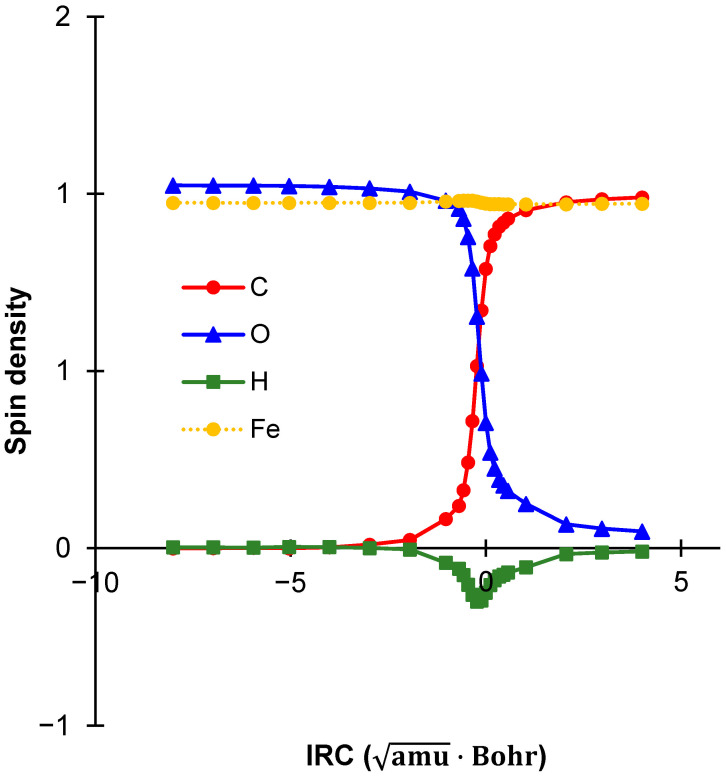
Spin densities on key atoms obtained from BOVB calculations, plotted against the IRC.

We next examined the evolution of spin densities along the IRC, as shown in Figure 5. On the reactant side of the coordinate, both Fe and O carried spin density values of approximately +1.0, while the abstracted hydrogen atom and the carbon atom exhibited negligible spin. This aligns well with the depiction in Figure 1a, where Fe and O maintain radical character, but the substrate remains largely unaffected. This spin distribution persisted up to the vicinity of the TS. Upon crossing the saddle point, dramatic shifts in spin densities were observed. The spin density on the oxygen atom decreased significantly, while that on the carbon atom rapidly increased (Figure 5), effectively reversing their spin populations. The decrease in spin density on the oxygen atom reflects the reduction of its initial radical character as the iron(IV)–oxo unit transitions into an iron(III)–hydroxide species. Simultaneously, the substrate is transformed into a methyl radical, thereby resulting in the emergence of substantial spin density on the carbon atom. During this transition, the C–H bond is cleaved, and a new O–H bond is formed. Additionally, near the saddle point, the spin density on the hydrogen fragment became slightly negative but quickly returned to zero. This indicates that the hydrogen atom does not develop strong radical character during the reaction. Throughout the process, the spin density on Fe remained stable at approximately +1.0, without notable fluctuation, supporting the notion that the unpaired electron on Fe (Scheme 3) does not actively participate in the reaction mechanism.

## 3. Materials and Methods

The geometries of the original DFT models (RC_0_, TS_0B_, and INT_0B_), which feature a porphine ring and HS^−^ as the equatorial and proximal ligands, respectively, were optimized at the B3LYP-D3(BJ)/6-31G* level of theory [53,54,55,56,57,58,59,60,61,62,63]. Following our previous studies [36,37,41], we extracted key atoms (Fe, O, and CH_4_) from the DFT-optimized geometries to construct the VB models RC_1_, TS_1B_, and INT_1B_. The VB models were reoriented such that the iron atom was placed at the origin and the Fe–O bond aligned along the *z*-axis. One of the Fe–O–N planes was aligned with the *xz* plane to ensure that all atoms in CH_4_ had positive *x*, *y*, and *z* coordinate values. Additionally, IRC calculations at the same level of theory were performed in both forward and reverse directions from TS_0B_. All DFT calculations were carried out using Gaussian 16 [64]. Selected geometries along the IRC were used to construct simplified models for VB calculations. In all VB calculations, an OEEF was consistently applied along the Fe–O axis, with a field strength of −0.115 au (Scheme 2).

After constructing the models, ab initio VB calculations were performed using XMVB 3.0 (GAMESS version, GAMESS 2022 R2) [65,66,67]. These calculations employed the 6-31G*(6D,10F) basis set and began with VBSCF calculations [68,69], in which the total wave function Ψ is expressed as a linear combination of VB structures {Φ*_K_*}, with both the orbitals within each structure and the coefficients *C_K_* optimized:

(1) $$\Psi =\underset{K}{\sum }C_{K}\Phi_{K}$$

Following the VBSCF calculations, BOVB calculations [70,71,72] were performed. This method allows the orbitals in different VB structures to “breathe”, i.e., to vary independently, thus providing a better description of dynamical electron correlation. Consistent with the protocol from our previous studies [36,37,41], only the active orbitals were allowed to vary (Figure 2), while all remaining orbitals were constrained to be common across all VB structures to reduce computational cost. VB weights were computed using the Chirgwin–Coulson formula [73]:

(2) $$W_{K}=C_{K}^{2}+\underset{L\neq K}{\sum }C_{K}C_{L}\left\langle \Phi_{K}|\Phi_{L}\right\rangle$$

All VB calculations were carried out using four active orbitals (Figure 2) and four active electrons (red dots in Scheme 3), which in principle generate over 20 VB structures. Among these, eight structures (Φ_1_–Φ_8_) were initially selected for inclusion in the VB calculations. However, Φ_7_ and Φ_8_ (Scheme S1) were found to contribute negligibly to the overall electronic structure and, in some cases, caused convergence issues. Therefore, these two structures were excluded from further analysis. Consequently, six VB structures (Φ_1_–Φ_6_) were retained for the final VB calculations, as shown in Scheme 3. VBSCF orbitals (Figure 2) were visualized using MacMolPlt v7.7.3 [74].

Because the geometries along the reaction coordinate lack high symmetry, the straightforward grouping of AOs for constructing VB orbitals, as employed in our previous work, was not applicable. Therefore, we defined three orbital groups corresponding to the Fe=O moiety, the abstracted hydrogen atom, and the CH_3_ fragment. This strategy was adopted to capture the essential electronic features of the system while reducing computational complexity.

## 4. Conclusions

We employed ab initio VB calculations to investigate the H-abstraction step in alkane hydroxylation catalyzed by P450s. To compensate for the reduced accuracy resulting from the use of a minimal iron(IV)–oxo model, an OEEF was applied to mimic the electronic effects of the equatorial porphyrin and proximal thiolate ligands. Among the six VB structures considered, two—corresponding to covalent C–H and O–H bonds—were found to play dominant roles in shaping the potential energy profile. In addition, two other VB structures featuring a cationic hydrogen fragment made substantial contributions to the wave function. Interestingly, although the total energy is highest at the TS, this region also exhibits the most extensive configuration mixing among VB structures, resulting in maximal resonance stabilization. Spin density analysis further supports the presence of significant electronic reorganization near the TS, consistent with C–H bond cleavage and O–H bond formation. These observations underscore the utility of VB theory in providing chemically intuitive insights into the origins of activation barriers. To improve the quantitative accuracy of VB results, future work will need to employ more realistic models that better reflect the native Cpd I species and biologically relevant alkane substrates. Nevertheless, this study offers a unique perspective on the H-abstraction step in P450 catalysis and serves as a valuable foundation, particularly by revealing features that are not readily captured by conventional MO-based approaches. Further applications of VB methods to P450s and other bioinorganic systems are expected to deepen our understanding of the intrinsic reactivity patterns of short-lived reactive species.

## Figures and Tables

**Table 1:** Relative energies (kcal/mol) of RC, TS, and INT for Path B, obtained from DFT, VBSCF, and BOVB calculations.

	RC	TS	INT
DFT ^a^	0.00	28.41	25.64
VBSCF ^b^	0.00	43.46	11.52
BOVB ^b^	0.00	39.69	10.19

^a^ Calculated for RC_0_, TS_0B_, and INT_0B_. ^b^ Calculated for RC_1_, TS_1B_, and INT_1B_.

**Table 2:** Weights (%) of VB structures Φ_1_–Φ_6_ (*W*_1_–*W*_6_) obtained from BOVB calculations for models RC_1_, TS_1B_, and INT_1B_. *W*_CH_ represents the sum of the weights of Φ_1_–Φ_3_, while *W*_OH_ is the sum of the weights of Φ_4_–Φ_6_. The most dominant structures are shown in bold.

Weight	RC_1_	TS_1B_	INT_1B_
*W*_1_	**68.30**	11.45	0.64
*W*_2_	4.27	1.85	0.16
*W*_3_	27.43	12.00	0.50
*W*_4_	−0.01	**35.98**	**57.34**
*W*_5_	0.00	3.66	3.48
*W*_6_	0.00	35.06	37.89
*W*_CH_	100.00	25.30	1.30
*W*_OH_	0.00	74.70	98.70

## Data Availability

Data are contained within the article and Supplementary Materials.

## Supplementary Materials

The following supporting information can be downloaded at: https://www.mdpi.com/article/10.3390/molecules30102242/s1, Additional VB structures and XYZ coordinates of models (PDF).
