# Supplementary material for: Valence Bond Insights into the H-Abstraction Barrier in Cytochrome P450
Source: Molecules. 2025 May 21;30(10):2242. doi: 10.3390/molecules30102242 (PMC12114163; doi:10.3390/molecules30102242)
Supplement: Supplementary file 1 [file molecules-30-02242-s001.zip › molecules-3621514-supplementary.pdf]

1. Additional VB structures

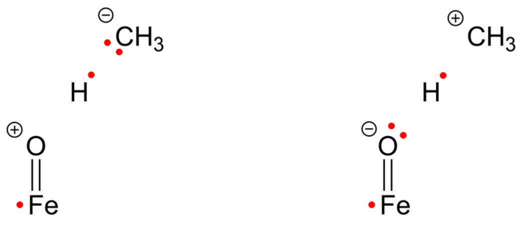

Φ_3 Φ_6

Scheme S1. Two additional VB structures, Φ_7 and Φ_8.

2. XYZ coordinates of models

* Values in parentheses indicate the total charge and spin multiplicity of each system.

=== Model RC_0 ===

E(B3LYP-D3BJ/6-31G*) =
-2766.64863759 au

(0,2)

Fe	9.226175	30.879082	2.209494
O	7.908343	30.056227	2.665557
H	5.884158	30.177913	1.447069
C	5.024838	30.325139	0.787758
H	5.179567	29.768615	-0.141545
H	4.114210	29.967006	1.277041
H	4.921431	31.388871	0.556176
S	11.223175	31.941264	1.082813
H	12.138757	30.986387	1.359425
N	9.643989	29.617994	0.684061
N	10.471038	29.836214	3.388000
N	9.079073	32.291405	3.614333
N	8.241658	32.067570	0.916070
C	9.112661	29.651970	-0.589128
C	9.539107	28.497258	-1.338492
C	10.324130	27.755197	-0.511562
C	10.375247	28.448742	0.750764
C	11.086280	28.645580	3.097906
C	11.800488	28.152044	4.249494
C	11.604734	29.056604	5.247542
C	10.772886	30.100076	4.701986
C	9.566108	32.233775	4.895964
C	9.164827	33.400509	5.641898
C	8.427682	34.169615	4.794788
C	8.379300	33.467411	3.536345
C	7.653520	33.279350	1.193579
C	6.948448	33.773232	0.036511
C	7.104199	32.841580	-0.942553
C	7.909966	31.783156	-0.385216
C	8.297107	30.650610	-1.083873
H	7.946415	30.550320	-2.105794
C	11.046771	27.997557	1.870189
H	11.581810	27.057033	1.786420
C	10.354280	31.215180	5.407946
H	10.670926	31.300217	6.442532
C	7.719480	33.935739	2.409102
H	7.207696	34.889250	2.490941
H	10.825418	26.818488	-0.717014
H	9.263631	28.298452	-2.365821
H	12.371011	27.232838	4.271081
H	11.981540	29.037837	6.261694
H	9.427535	33.590552	6.674217
H	7.955555	35.124601	4.984260
H	6.410205	34.711152	-0.000835
H	6.720163	32.851881	-1.953991

=== Model TS_{0A} ===

E(B3LYP-D3BJ/6-31G*) =
-2766.61544376 au

(0,2)

Fe	9.246784	30.978202	2.175597
O	7.740654	30.245604	2.675297
H	6.880058	30.294301	1.885557
C	5.872697	30.268861	1.000838
H	5.622474	31.316305	0.846141
H	6.316215	29.776333	0.137970
H	5.115958	29.686789	1.524519
S	11.318924	31.893843	1.753158
H	11.893333	30.822011	1.164944
N	9.553956	29.624248	0.738428
N	10.137288	29.761542	3.512023
N	9.090310	32.401131	3.579291
N	8.386765	32.188111	0.848534
C	9.285423	29.761132	-0.602956
C	9.614180	28.543658	-1.298275
C	10.060727	27.661376	-0.361109
C	10.026671	28.342364	0.907437
C	10.520331	28.461629	3.307178
C	11.084999	27.916343	4.518794
C	11.055740	28.910018	5.447557
C	10.455764	30.058747	4.811959
C	9.562204	32.341072	4.868801
C	9.201525	33.542157	5.579866
C	8.482337	34.311637	4.717518
C	8.419450	33.592413	3.469779
C	7.810921	33.412555	1.103701
C	7.295472	33.977664	-0.116957
C	7.601420	33.105955	-1.117869
C	8.282752	31.992465	-0.510583
C	8.718101	30.875141	-1.199225
H	8.566584	30.852031	-2.273048
C	10.453984	27.785890	2.101002
H	10.802977	26.758978	2.078720
C	10.218895	31.267130	5.443235
H	10.529372	31.363866	6.478094
C	7.794714	34.060020	2.325947
H	7.302808	35.025382	2.378170
H	10.394071	26.641045	-0.498468
H	9.497957	28.396933	-2.364107
H	11.462540	26.907136	4.618726

H	11.397493	28.887317	6.474013
H	9.460333	33.743243	6.611114
H	8.033146	35.281075	4.888943
H	6.790618	34.932533	-0.183233
H	7.390939	33.190780	-2.175942

=== Model TS$_{0B}$ ===

E(B3LYP-D3BJ/6-31G*) =
-2766.60336071 au

(0,2)

Fe	9.257635	30.873157	2.015072
O	7.812322	29.977337	2.419925
C	5.635066	31.265512	2.091965
H	6.890882	30.558599	2.272703
H	5.790982	32.148829	2.706139
H	5.592896	31.441919	1.020384
H	4.927291	30.536578	2.480309
S	11.392769	31.846931	1.956701
H	12.109649	30.804544	1.481700
N	9.757037	29.547015	0.597416
N	10.177666	29.682765	3.392643
N	8.921380	32.258101	3.419099
N	8.417177	32.051234	0.675222
C	9.372736	29.588750	-0.717085
C	9.849831	28.423332	-1.419975
C	10.524608	27.668591	-0.509393
C	10.445879	28.368399	0.749521
C	10.804584	28.480766	3.181396
C	11.264573	27.927571	4.435267
C	10.890153	28.801543	5.407182
C	10.215677	29.896460	4.745634
C	9.153100	32.120937	4.769819
C	8.751234	33.318456	5.465102
C	8.289629	34.187881	4.524162
C	8.388621	33.513574	3.254412
C	7.941163	33.331528	0.849056
C	7.396105	33.839181	-0.385317
C	7.548285	32.857111	-1.314942
C	8.206496	31.756381	-0.654547
C	8.634375	30.609087	-1.299620
H	8.399903	30.514694	-2.355224
C	10.952977	27.876920	1.944720
H	11.472837	26.924715	1.910344
C	9.723122	31.020162	5.388731
H	9.828512	31.061130	6.468283
C	7.950551	34.029984	2.044942
H	7.549740	35.038474	2.042212
H	11.021940	26.718430	-0.654156
H	9.681090	28.225614	-2.470443
H	11.794429	26.989459	4.536762
H	11.052737	28.734853	6.474996
H	8.830057	33.461681	6.534849
H	7.904783	35.190069	4.660270
H	6.958056	34.821483	-0.504648
H	7.269327	32.867227	-2.360511

=== Model INT$_{0A}$ ===

E(B3LYP-D3BJ/6-31G*) =
-2766.62441913 au

(0,2)

Fe	0.017341	-0.131287	-0.127618
O	-1.578641	-0.917836	0.029211
H	-2.180581	-0.388640	0.596256
C	-3.837296	0.664143	1.442610
H	-3.336983	1.021373	2.333994
H	-3.859865	1.289773	0.558833
H	-4.450413	-0.228116	1.484565
S	2.098591	0.803828	-0.276795
H	1.891137	1.620944	-1.332340
N	0.499756	-1.505463	-1.500918
N	0.837773	-1.265967	1.305128
N	-0.511961	1.232113	1.253548
N	-0.632512	1.094960	-1.573966
C	0.250099	-1.444863	-2.848645
C	0.738498	-2.637935	-3.496709
C	1.304651	-3.407493	-2.528537
C	1.154421	-2.691824	-1.284883
C	1.457412	-2.480620	1.137072
C	1.869234	-3.004333	2.414673
C	1.469576	-2.107199	3.357299
C	0.821710	-1.026591	2.658160
C	-0.336616	1.133279	2.614651
C	-0.808613	2.331419	3.260178
C	-1.235126	3.170397	2.274757
C	-1.037610	2.482860	1.025116
C	-1.166038	2.353553	-1.418719
C	-1.572150	2.877812	-2.697505
C	-1.305398	1.914617	-3.622523
C	-0.725780	0.802287	-2.914467
C	-0.326961	-0.379215	-3.515129
H	-0.459446	-0.467271	-4.588122
C	1.619767	-3.146964	-0.064814
H	2.128125	-4.104895	-0.044279
C	0.264665	0.078232	3.277702
H	0.331735	0.135941	4.358799
C	-1.341478	3.014969	-0.215295
H	-1.763045	4.013993	-0.246062
H	1.786339	-4.371645	-2.624994
H	0.659701	-2.833734	-4.558032
H	2.384005	-3.946131	2.552099
H	1.590623	-2.155071	4.431546
H	-0.786418	2.500038	4.328892

H	-1.643214	4.168458	2.366238
H	-2.015514	3.853825	-2.845486
H	-1.480976	1.935063	-4.690103

=== Model INT_{0B} ===

E(B3LYP-D3BJ/6-31G*) = -2766.60776978 au

(0,2)

Fe	-0.016221	-0.109502	-0.121611
O	-1.539903	-1.001873	0.197027
C	-3.935977	0.864881	0.986162
H	-2.238756	-0.364754	0.449131
H	-3.483401	1.269088	1.882949
H	-3.844508	1.403610	0.051337
H	-4.591968	0.005395	1.050542
S	2.100515	0.955709	-0.055801
H	2.911943	-0.086257	-0.345594
N	0.527886	-1.437347	-1.506241
N	0.892659	-1.249605	1.280350
N	-0.469131	1.266479	1.274341
N	-0.787889	1.088329	-1.505572
C	0.180162	-1.406997	-2.832782
C	0.696761	-2.568768	-3.518669
C	1.370446	-3.300260	-2.590428
C	1.247735	-2.590419	-1.338080
C	1.573937	-2.430016	1.097428
C	2.001653	-2.965216	2.368411
C	1.539948	-2.115748	3.325267
C	0.848460	-1.048329	2.640309
C	-0.327665	1.121069	2.633022
C	-0.787163	2.308021	3.314059
C	-1.176126	3.189486	2.350944
C	-0.982608	2.528130	1.084279
C	-1.271626	2.364531	-1.352623
C	-1.758313	2.874700	-2.610278
C	-1.573745	1.885890	-3.528867
C	-0.954926	0.784501	-2.833118
C	-0.525851	-0.384800	-3.447589
H	-0.726237	-0.488073	-4.509573
C	1.761038	-3.044499	-0.128581
H	2.312428	-3.979537	-0.141049
C	0.252972	0.031938	3.268541
H	0.291664	0.056536	4.353207
C	-1.329593	3.055296	-0.149128
H	-1.726465	4.065417	-0.170247
H	1.897147	-4.236837	-2.718676
H	0.558597	-2.774785	-4.572024
H	2.564382	-3.881128	2.492908
H	1.650792	-2.185791	4.399491
H	-0.782486	2.441729	4.387968
H	-1.567292	4.191380	2.470224
H	-2.187213	3.858084	-2.751866
H	-1.813535	1.891124	-4.584116

=== Model RC_1 ===

(2,3)

Fe	0.000000	0.000000	0.000000
O	0.000000	0.000000	1.619218
H	1.014099	1.846617	2.706057
C	1.501222	2.762186	3.051368
H	2.549538	2.755370	2.738223
H	0.999351	3.628818	2.611606
H	1.448652	2.824006	4.142334

=== Model TS_{1B} ===

(2,3)

Fe	0.000000	0.000000	0.000000
O	0.000000	0.000000	1.747950
H	0.477511	0.892050	2.177859
C	1.085753	2.048448	2.812092
H	2.080072	2.041094	2.372726
H	0.445840	2.872729	2.508358
H	1.028414	1.765737	3.860848

=== Model INT_{1B} ===

(2,3)

Fe	0.000000	0.000000	0.000000
O	0.000000	0.000000	1.794286
H	0.776578	0.501524	2.115647
C	2.396729	1.597109	3.040724
H	3.182870	0.986485	2.614630
H	2.082653	2.498274	2.529107
H	2.026757	1.386412	4.036672
